# Improving student-perceived benefit of academic advising within education of occupational and physical therapy in the United States: a quality improvement initiative

**DOI:** 10.3352/jeehp.2017.14.4

**Published:** 2017-03-25

**Authors:** Lisa J. Barnes, Robin Parish

**Affiliations:** 1 Department of Physical Therapy, School of Health Related Professions, The University of Mississippi Medical Center, Jackson, MS, USA; 2 Department of Occupational Therapy, School of Health Related Professions, The University of Mississippi Medical Center, Jackson, MS, USA; Hallym University, Korea

**Keywords:** Health occupations, Health personnel, Quality improvement, Student, United States

## Abstract

Academic advising is a key role for faculty in the educational process of health professionals; however, the best practice of effective academic advising for occupational and physical therapy students has not been identified in the current literature. The purpose of this quality improvement initiative was to assess and improve the faculty/student advisor/advisee process within occupational and physical therapy programs within a school of allied health professions in the United States in 2015. A quality improvement initiative utilizing quantitative and qualitative information was gathered via survey focused on the assessment and improvement of an advisor/advisee process. The overall initiative utilized an adaptive iterative design incorporating the plan-do-study-act model which included a three-step process over a one year time frame utilizing 2 cohorts, the first with 80 students and the second with 88 students. Baseline data were gathered prior to initiating the new process. A pilot was conducted and assessed during the first semester of the occupational and physical therapy programs. Final information was gathered after one full academic year with final comparisons made to baseline. Defining an effective advisory program with an established framework led to improved awareness and participation by students and faculty. Early initiation of the process combined with increased frequency of interaction led to improved student satisfaction. Based on student perceptions, programmatic policies were initiated to promote advisory meetings early and often to establish a positive relationship. The policies focus on academic advising as one of proactivity in which the advisor serves as a portal which the student may access leading to a more successful academic experience.

## Introduction

Academic advising is a key role for faculty in the educational process of health professionals; however, the best practice of effective academic advising for occupational and physical therapy students has not been identified in the current literature. Individual student perception of the benefit inherent in the advisor/advisee relationship should be considered in the development of best practice. Current data extrapolated from nursing and medical education suggest that the advisor/advisee relationship may play a critical role in the development of academic skills, personal growth and professionalism among students across the health professions spectrum [1-4]. A link between effective advising and best practices from both the teaching and learning poles of education has been identified [5]. Students strongly seek a caring, authentic, available, knowledgeable advisor to serve as a compass and portal to help facilitate their journey towards becoming successful healthcare professionals in the transformational healthcare system of the future [2].

**Fig. 1.**
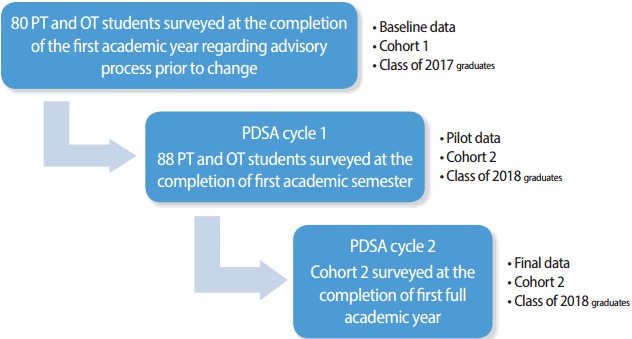
Quality improvement process over time. PT, physical therapy; OT, occupational therapy; PDSA, plan-do-study-act model.

The role of academic advising in health professional education differs from that in general undergraduate education in that the advisory process extends beyond course planning for individual degrees, and provides ongoing support for academic success [3,5]. The purpose of this quality improvement initiative was to assess and improve the faculty/student advisor/advisee process within entry-level occupational and physical therapy academic programs within a school of allied health professions, the University of Mississippi Medical Center, the United States in 2015. The academic advisor process was identified as an area of improvement within the educational program because it had become more of a reactive process that intervened when students found themselves in academic difficulties. Advisors were assigned small groups of advisees, but had no defined protocols to guide advisory interaction. The quality improvement project explained here was undertaken to create an advisor/advisee process which was proactive in serving the academic needs of students in order to promote academic success which facilitates the development of professionalism. The overall initiative utilized an adaptive iterative design incorporating the plan-do-study-act (PDSA) model which included a three-step process over a one year time frame utilizing 2 cohorts of the first with 80 students and the second with 88 students as defined in Fig. 1.

## Baseline

### Baseline method

In order to understand student perceptions of the original advisory process, baseline information was gathered by administering a questionnaire developed by the authors to physical therapy and occupational therapy students at the University of Mississippi Medical Center who had just completed a first full academic year in their respective programs in 2015. The questionnaire included opinion items, yes/no questions, and open ended questions to be answered by the participants. One Likert-type item was included to gather information on student-perceived benefit (Appendix 1). The authors systematically went through each participant’s qualitative comments and analyzed frequencies of words to discover the emerging themes. These themes are reflected in the results. Statistical analysis was conducted using IBM SPSS for Windows ver. 24.0 (IBM Corp., Armonk, NY, USA). Correlations were calculated using Spearman’s correlation coefficient, rs, due to the nature of the variables with one being ordinal and the other continuous.

### Baseline results

Eighty students (94.1%) participated in the baseline survey out of total 85 subjects. At baseline, 63 students (78.8%) could name his or her academic advisor; however, 50 students (62.5%) had never had an advisory meeting. Only 14 students (17.5%) had met with advisors within the first month of starting the academic program, and those who did not meet with advisors overwhelmingly reported that they felt getting to know the advisor early would have been beneficial (75.8 %).

Students were asked to respond to the statement: “The advisor/advisee mentoring relationship has been beneficial to my overall growth as a student of the health professions.” Only 21 (26.3%) agreed or strongly agreed, and these students reported meeting more than 37 combined times with advisors during the first academic year. Forty-one students (51.3%) were neutral on the issue and had met 16 combined times. Sixteen (20%) disagreed or strongly disagree and had met six combined times during the year. This seemed to indicate that more meetings with advisors led to greater benefit for the students. Statistical analysis showed that there was a significant relationship between the number of face-to-face meetings and the level of perceived benefit of the advisory program (r_s_= 0.40, P< 0.01).

Fifty seven participants (71.3%) reported developing a mentoring relationship with faculty members who were not assigned as academic advisors. When asked what characteristics students identified that made these faculty members seem approachable, the responses were categorized into the following themes: availability to the students; attitude that was perceived as helpful, kind, caring, respectful of the students, trustworthy, and encouraging; and, invested in the whole person (academic and personal).

Students were asked to provide suggestions on how the advisor/advisee process might be made more beneficial, and the categories of responses were weighted as the following: early initial meetings, and regularly scheduled meetings thereafter (36.3%); a conscious effort to build a relationship as a mentor and guide in whom the student can confide for support and help when needed (21.3%); and, a clear definition of the advisory program (7.5%). Given these baseline findings of student perceptions, the quality improvement initiative using the PDSA model was begun with the first cycle being the pilot [6]. According to our institutional policy, institutional review board approval was not required for quality improvement activities.

## Cycle 1 (pilot)

The PDSA model calls for implementing small systematic changes in processes followed by outcomes assessment before proceeding to a broader scope of procedural transformation [6]. The first step in the quality improvement effort included a small number of faculty advisors who agreed to participate in a pilot of the proposed new process. The proposal was to incorporate an initial meeting with student advisees within the first two weeks of entering the academic program, a follow-up meeting within the first semester, and a meeting at least once per semester thereafter. Six advisors with a total of 29 student advisees took part in the pilot; however, all academic advisors and students were actively involved in the usual advisory process during the semester.

### Cycle 1 method

In the pilot semester, the participating advisors followed the proposed schedule and tracked the actions and responses from advisees. Each advisor scheduled an individual face-to-face meeting with each student advisee during the first 2 weeks of school to serve as an introductory meeting in which they would explain that the advisor role was for mentoring and growth, and was not just for times of academic difficulty.

A second individual meeting was schedule later in the semester with students prepared to discuss personal strengths and opportunities for improvement that had been identified during the first several weeks of school. Faculty advisors were encouraged to use their own individual communication styles as they establish relationships with the advisees.

Upon completion of the semester a survey was conducted of the students completing their first academic semester in the programs. Eighty-eight (97.8%) out of 90 students participated in the survey. Although only 29 students had been assigned to the participating advisors, student communication among their peers led to unexpected improvement in the advisory process across the entire cohort.

### Cycle 1 results

At the end of only one academic semester, 87 (98.9%) the students could name his or her academic advisor, and 66 students (75.5%), up from 17.5% at baseline, reported meeting with advisors within the first month of school. Of the 22 students (25.0%) reporting that they did not meet their academic advisor during the first month of school, 52 students (59.1%) reported that getting to know the advisor early would have been beneficial for them.

When these students were asked to respond to the statement: “The advisor/advisee program has been beneficial to my overall first semester experience.” Sixty-one (69.3%) agreed or strongly agreed, and these students represented more than 89 combined meetings with advisors. Twenty-three students (26.1%) were neutral on the issue with only 11 combined meetings, and four (4.6%) disagreed or strongly disagree with the statement; however, none of these participants had met with their advisors. Once again these findings indicated that increasing the number of meetings with academic advisors was significantly correlated with greater perceived benefit by the students (r_s_= 0.59, P< 0.01).

## Cycle 2

Given the positive results, in the following semester the new process was introduced to the entire group of faculty advisors for incorporation as the new advisory process within two health professional academic programs.

### Cycle 2 method

With participation across the programs, a follow-up survey was completed at the end of the first full academic year for those students who were the first to experience the new process. Eighty-eight students participated in the survey which served as the final evaluation of the newly incorporated educational advisory process.

### Cycle 2 results

**Fig. 2.**
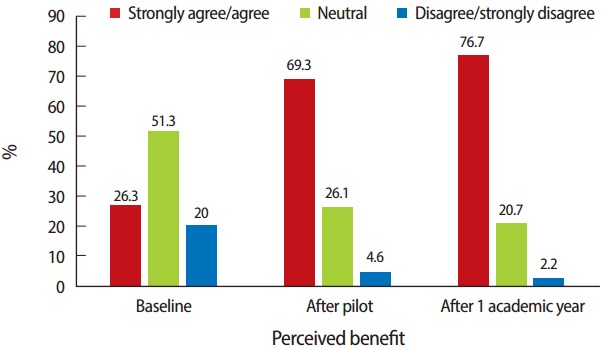
Perceived benefit of the advisor/advisee relationship. Baseline: 80 students surveyed at the end of first academic year experiencing the original process. After pilot: 88 students surveyed at the end of their first academic semester with new process. After academic year 1: same 88 students after one year with new process.

Results indicated that all students could name his or her academic advisor (up from 78.8% at baseline), and 68 students (77.3%) reported meeting with academic advisors early in the program, up from 17.5%. The findings revealed an improvement in the advisee’s perception of the benefit of the advisory program. At baseline, 26.3% found the process beneficial to their growth as a student of the health professions; however, the final results showed that after one year with the new process, 76.7% found it to be beneficial (Fig. 2).

**Fig. 3.**
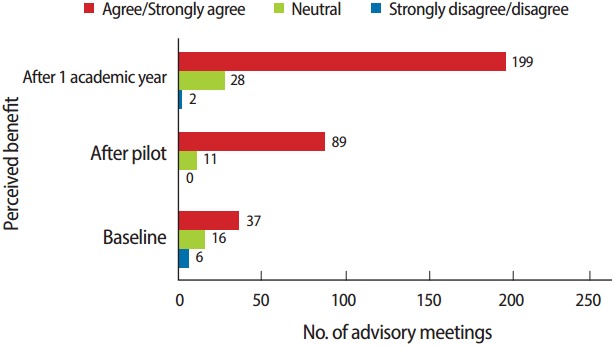
Number of meetings with advisors compared to perceived benefit of the advisor/advisee program. Baseline: 80 students surveyed at the end of first academic year experiencing the original process. After pilot: 88 students surveyed at the end of their first academic semester with new process. After academic year 1: same 88 students after one year with new process.

There was a significant correlation between the number of visits with advisors and the students’ perception of the benefit of the program (r_s_= 0.60, P< 0.01). This positive correlation was seen at each level of the initiative. As the number of meetings with advisors increased the perception of benefit also increased (Fig. 3). Raw data were available from Supplement 1.

Once again the students were asked if they had developed mentoring relationships with faculty members other than the assigned advisors. This category resulted in a similar level to baseline indicating that over time students tend to gravitate to individuals with whom they feel a natural ability to communicate. When asked what characteristics students found in these “unofficial” mentors, the responses were identical to baseline. The participants explained that the characteristics of availability, investment in the whole person, and a caring and helpful attitude made the faculty members approachable.

## Conclusion

Defining an effective advisory program with an established framework for interaction led to improved awareness and participation by students and faculty. Early initiation of the academic advisory process combined with increased frequency of interaction with focus on the personal and professional growth in addition to academic performance led to improved student satisfaction. Although this initiative focused solely on student perception, future studies should consider faculty perceptions of the program as well as objective measurements of academic successes that may be directly related to a proactive advisory process. This quality improvement initiative was specific to our institution. It is not intended to be generalizable, but rather an example of a successful quality improvement initiative to improve student perception of benefits of the advisory process.

Based on student perceptions within this quality improvement project, programmatic policies and procedures have been initiated to promote advisory meetings early and often to establish a positive relationship. The new policies place a focus on ensuring that the academic advisory process is one of proactivity in which the faculty advisor serves as a portal which the student may access leading to a more successful academic experience. Additionally, the policies allow for autonomy and individuality of the advisors/advisees in order to optimize the benefit of the interaction.

## Supplementary materials

Supplement 1. Data file is available from  https://dataverse.harvard.edu/dataset.xhtml?persistentId=doi:10.7910/DVN/OYEDE0

Supplement 2. Audio recording of the abstract.

## Appendix 1. Questionnaire use for the survey to occupational and physical therapy students

Advisor/Advisee relationships

Please circle: OT (occupational therapy) / PT (physical therapy)

1. What is the name of your academic advisor?      

 (If you do not know, please write “I do not know”)

2. Over the last year, how many face to face meetings have you had with your advisor, outside of class?

 a. 0

 b. 1

 c. 2

 d. 3

 e. 4+

3. Did you have an initial face-to-face meeting with your advisor during the first month of your first semester as a student?

 a. Yes

 b. No

 If you answered no, do you think this would have been beneficial to you?

 If you answered yes, explain if this was beneficial to you and how.

4. My meetings with my advisor were due to      (Please circle all that apply)

 a. Positive curricular discussions

 b. Discussions about academic difficulties

 c. Casual “getting-to-know-you” conversations

 d. I have never met with my advisor

 e. Others. Please explain         

5. Were your meetings with your advisor beneficial in establishing a professional mentoring relationship?

 a. Yes

 b. No

 c. I have never met with my academic advisor

 If yes, or no, please elaborate on your answer:

6. Have you established mentoring relationships with faculty members, who are not your assigned academic advisor?

 a. Yes

 b. No

 If you answered yes, what characteristics made this faculty member approachable?

Please rate the follow question:

The advisor/advisee mentoring relationship has been beneficial in my overall growth as a student of the health professions.

 0. Strongly disagree

 1. Disagree

 2. Neutral

 3. Agree

 4. Strongly agree

 Please give us any feedback on the subject of advisor/advisor relationships and how we can better benefit you in this area:
